# Multiscale Analysis of CFRP Laminates with MMF3 Criterion under Different Off-Axis Loading Conditions

**DOI:** 10.3390/ma11112255

**Published:** 2018-11-12

**Authors:** Zhun Liu, Zhidong Guan, Riming Tan, Jifeng Xu, Xing Li

**Affiliations:** 1 School of Aeronautic Science and Engineering, Beihang University, Beijing 100191, China; zhunliu@139.com (Z.L.); tanriming_mean@126.com (R.T.); 2 Beijing Aeronautical Science and Technology Research Institute, Beijing 102211, China; xujifeng@comac.cc (J.X.); lixing2@comac.cc (X.L.)

**Keywords:** multiscale analysis, carbon fiber reinforced plastic (CFRP) laminates, off-axis loading, thermal residual stress, constituent level

## Abstract

A multiscale analysis strategy with physical modified-micromechanics of failure (MMF3) criterion was proposed to analyze the failure behaviors of carbon fiber reinforced plastic (CFRP) laminates. The quantitative relationship between the macro- and micro- stresses was determined considering two typical fiber distributions. Thermal residual stress was taken into account in the stress transformation. The failures were defined and the properties of damaged elements were degraded at the constituent level. The back-calculation method based on the iteration algorithm was proposed to determine the micro strength with macro mechanical tests. A series of off-axis loading tests were conducted to verify the established multiscale models. The predicted strength was also compared with the results using micromechanics of failure (MMF) criterion to present accuracy improvements. Thermal residual stress was found to affect the strength by contributing to the matrix damage status. Meanwhile, sensitivity analysis was provided for the matrix-dominant micro strength to investigate its physical meaning. Results suggest that the micro tensile and compressive strength of the matrix influenced the off-axis tensile and compressive strengths respectively, with relative large off-axis angles, while the micro shear strength of the matrix dominated when the off-axis angles were relative small.

## 1. Introduction

Carbon fiber reinforced plastics (CFRPs) have been developed and optimized over the past 50 years. Before practical application, the composite structures must be evaluated comprehensively to ensure their integrity. Ideally, the mechanical behaviors can be predicted with minimum cost in through fully-developed structural analysis theories and models [1]. The early proposed failure theories like Tsai-Wu tensor criterion [2] etc. are usually in conjunction with phenomenological models to predict the strength of composites. These semi-empirical methods assume that the composites are homogeneous in the single ply. Failures are defined based on stress–strain data at the macro level. They are widely used in the engineering because of their usability.

In order to further explain the failure mechanisms of composites, many recent studies focus on the mechanical behaviors of composites at the constituent level. Some non-empirical theories like Hashin criterion [3], Puck’s action plane strength criterion [4], LaRC03 criterion [5] etc. have emerged. These failure theories still use macro-level stresses when considering the failure of constituents. Failure characterization becomes more complex and in general requires one or more additional interactive parameters. In the micromechanics-based models proposed by Chamis et al. [6], failures were defined using the stress-based modified distortion energy (MDE) of laminates. The macro effective strength of laminates in the MDE was determined through the homogenized strength of constituents.

Since the macro-level failure mechanisms are the consequence of an accumulation of micro-level events, it is more intuitive to determine the failures aimed at the constituents (fiber and matrix) directly. One way is to establish the models with fully-detailed microstructures [7,8]. This is obviously inefficient in modeling and numerical computation. Another way is to characterize the properties of composites based on various representative volume elements (RVEs). Composites with periodic and random distributions of fiber were studied by Bouaoune [9] and Beicha [10] via RVEs. The periodic RVE can be used to capture global effective elastic properties, but the microstructures affect the effective properties of composites with high fiber fractions. Therefore, a more sensible multiscale approach is to combine macro models with the RVE models. This takes advantage of the efficiency of macro models and the accuracy of the micro models [11,12].

Typically, the strain invariant failure theory (SIFT) was proposed based on the micro strains by Gosse et al. [13]. Tay et al. [14] combined the element-failure method (EFM) with the SIFT to study the damage progression of composites. In the study of long-term, open-hole compression strength of laminates from Cai et al. [15], micro-level failures were also addressed with the SIFT. However, the critical values in the SIFT need to be determined by tests of the laminates with specially-designed stacking sequences.

Stier et al. [16] studied the notched and un-notched laminates based on a multiaxial mixed model continuum damage model (MMCDM). A 3-D extension of Hashin criterion was used to determine damage initiation based on the average strains within the RVE. Mayes et al. [17,18] proposed the multi-continuum theory (MCT) to define the failure of composites using the average stresses within the constituents. The changes in micro-level properties were considered among the failure criterion. Due to the homogenization of the RVE or constituents, it is difficult to distinguish damage initiation and degradation, due to localized peak stresses in the fiber or matrix using the MMCDM or MCT.

Ha et al. proposed micromechanics of failure (MMF) criterion to predict the ultimate strength of composite laminates [19,20]. The failures of fiber and matrix were determined by a maximum stress criterion and a modified von Mises failure criterion respectively. With the MMF and RVEs, Muthusamy [21] and Huang et al. [22,23] studied the failure envelopes of composites under different loading conditions. Besides strength prediction, some multiscale models with MMF criterion have been established to study the failure and life of composites [24,25,26,27].

However, the predicted results with MMF criterion revealed two significant drawbacks: (1) premature matrix failure under longitudinal tension, and (2) lower predicted shear strength. Sun et al. [28] circumvented these problems by modifying the mechanical amplification factors to compensate for the shear effect. Better results have been obtained, but correction coefficients need to be determined by the matrix tensile failure strain and the ply in-plane shear strength. The process of numerical calculation also becomes more complicated. Afterwards, the modified micromechanics of failure (MMF3) criterion were elaborated to improve the prediction accuracy by Sihn S. [29] The micro shear strength of the matrix was considered independent of the tensile and compressive strength in the MMF3 criterion. This means that the properties of the matrix in the composites are not the same as those of the pure resin. Moreover, the longitudinal tensile micro stress contributed only to the failure of the fiber, rather than the matrix.

In this context, the present study intends to develop a multiscale strategy taking thermal residual stress into account, defining damage initiation with MMF3 criterion, and degrading the properties of damaged elements at the constituent level directly. The main modification of the micromechanics-based criterion has been focused on the definition of matrix damage. Therefore, multiscale models were established to analyze the matrix-dominant mechanical behaviors of unidirectional laminates under off-axis loading. The off-axis tensile and compressive tests with various off-axis angles were conducted to comprehensively assess the effectiveness of the developed strategy. The influence of thermal residual stress on the off-axis strength was explained via RVE models. Additionally, sensitivity analyses were performed to investigate the physical meanings of the matrix-dominant micro strength in the MMF3 criterion.

## 2. Theory and Approach

### 2.1. Stress Transformation

In order to determine the failure modes at the micro level, the micro stresses in the fiber and matrix can be obtained by FE analyses of RVEs. The quantitative relationship between the macro stresses and the micro stresses can be written using the stress amplification factors [30]:

(1) $$\sigma =M_{\sigma }\bar{\sigma }+A_{\sigma }\Delta T$$

where σ, $\bar{\sigma }$ (6 × 1) are the micro and macro stress vectors respectively, $M_{\sigma }$ (6 × 6) is the matrix of mechanical stress amplification factor caused by the discrepant mechanical properties of fiber and matrix, and $A_{\sigma }$ (6 × 1) is the matrix of thermal stress amplification factor caused by the different thermal expansion coefficients. Since the coupling effect will not change the values of the two micro longitudinal shear stresses, Equation (1) can be deduced as:

(2) $$\left\{ \begin{array}{c} \sigma_{11}\\ \sigma_{22}\\ \sigma_{33}\\ \sigma_{12}\\ \sigma_{13}\\ \sigma_{23} \end{array}\}=[\begin{array}{cccccc} M_{11} & M_{12} & M_{13} & 0 & 0 & M_{16}\\ M_{21} & M_{22} & M_{23} & 0 & 0 & M_{26}\\ M_{31} & M_{32} & M_{33} & 0 & 0 & M_{36}\\ 0 & 0 & 0 & M_{44} & M_{45} & 0\\ 0 & 0 & 0 & M_{54} & M_{55} & 0\\ M_{61} & M_{62} & M_{63} & 0 & 0 & M_{66} \end{array}]\{\begin{array}{c} {\bar{\sigma }}_{11}\\ {\bar{\sigma }}_{22}\\ {\bar{\sigma }}_{33}\\ {\bar{\sigma }}_{12}\\ {\bar{\sigma }}_{13}\\ {\bar{\sigma }}_{23} \end{array}\}+[\begin{array}{c} A_{11}\\ A_{21}\\ A_{31}\\ 0\\ 0\\ A_{61} \end{array}]\Delta T\right.$$

**Figure 1:**
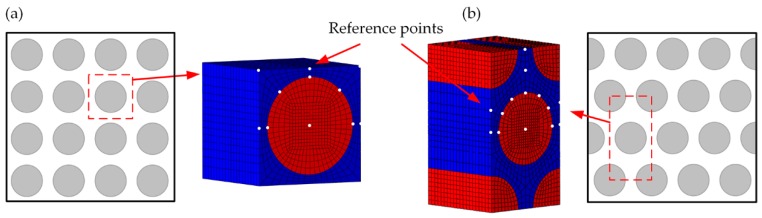
Representative volume elements: (**a**) Square RVE; (**b**) Hexagon RVE.

Two typical distributions of fiber and matrix are considered, as shown in Figure 1. Square and hexagon RVEs are used to obtain the stress amplification factors. Reference points are chosen to be the maximum stress points under different loading cases, which can cover the dangerous points in the calculation. Mechanical and thermal stress amplification factors need to be calculated at each reference point of the RVEs [31].

### 2.2. Failure Characterization

In MMF criterion, the failures of the fiber and matrix are determined by the maximum longitudinal stress and modified von Mises stress:

Fiber failure:

(3) $$\sigma_{11}=T_{f}\;\mathrm{or}\;\sigma_{11}=C_{f}\;$$

Matrix failure:

(4) $$\left(\frac{1}{T_{m}}-\frac{1}{C_{m}}\right)I_{1}+\frac{\sigma_{VM}^{2}}{C_{m}T_{m}}=1$$

where *T_f_* and *C_f_* are tensile and compressive strength of fiber respectively, *T_m_* and *C_m_* are tensile and compressive strength of the matrix, respectively. The first stress invariant *I*_1_ and the von Mises equivalent stress σ_VM_ can be expressed as:

(5) $$\begin{array}{l} I_{1}=\sigma_{11}+\sigma_{22}+\sigma_{33}\\ I_{2}=\sigma_{11}\sigma_{22}+\sigma_{22}\sigma_{33}+\sigma_{11}\sigma_{33}-(\tau_{12}^{2}+\tau_{13}^{2}+\tau_{23}^{2})\\ \sigma_{VM}=\sqrt{I_{1}^{2}-3I_{2}} \end{array}$$

The reason why the shear strength is usually underestimated in the MMF criterion is that there are only two critical values (*T_m_*, *C_m_*) determining the failure behavior of the matrix.

For an isotropic bulk resin, the shear strength $S_{m}^{bulkresin}$ can be calculated by the two parameters given above:

(6) $$S_{m}^{bulkresin}=\sqrt{\frac{T_{m}C_{m}}{3}}$$

However, 3 independent parameters, transverse tensile strength $Y_{T}$, transverse compressive strength $Y_{C}$ and in-plane shear strength S, are usually needed to characterize the matrix-dominant failure behavior at the macro level. This indicates that the micro in-situ shear strength of the matrix in a laminate may not be equal to that given in Equation (6). An independent shear strength of the matrix should be defined to characterize the failure behavior at the micro level.

According to Equation (6), the matrix failure criterion in Equation (4) can be derived as:

(7) $$\left(\frac{1}{T_{m}}-\frac{1}{C_{m}}\right)I_{1}+\frac{1}{T_{m}C_{m}}I_{1}^{2}-\frac{1}{S_{m}^{2}}I_{2}=1$$

The other problem is that when the longitudinal tensile performance is predicted using MMF criterion, the matrix fails before the fiber, satisfying the failure criterion. This causes a lower predicted longitudinal tensile strength. To solve this problem, it is believed that the micro stress $\sigma_{11}$ will not affect the failure behavior of the matrix since the longitudinal load is mostly carried by the fiber. To summarize, in total, there are 5 independent micro strength dominating the fiber and matrix failures in the MMF3 criterion; the matrix failure criterion can therefore be written by stress terms:

(8) $$\left(\frac{1}{T_{m}}-\frac{1}{C_{m}}\right)\left(\sigma_{22}+\sigma_{33}\right)+\frac{1}{T_{m}C_{m}}{\left(\sigma_{22}+\sigma_{33}\right)}^{2}-\frac{1}{S_{m}^{2}}\left[\sigma_{22}\sigma_{33}-\left(\tau_{12}^{2}+\tau_{13}^{2}+\tau_{23}^{2}\right)\right]=1$$

### 2.3. Damage Evolution

In the present study, the progressive damage analysis was actualized by degrading the properties at the constituent level. Once the stress states of fiber or matrix satisfy the failure criterion in Equation (3) or (8), the corresponding constituent properties should be degraded. $D_{f}=0.01$ and $D_{m}=0.05$ were taken as the degradation factors for the fiber and matrix damage. The exact degradation principles are:

Fiber failure:

(9) $$\begin{array}{l} E_{1f}^{FF}=D_{f}E_{1f}^{IN},\;E_{2f}^{FF}=E_{2f}^{IN},\;E_{3f}^{FF}=E_{3f}^{IN}\\ \nu_{12f}^{FF}=D_{f}\nu_{12f}^{IN},\;\nu_{13f}^{FF}=D_{f}\nu_{13f}^{IN},\;\nu_{23f}^{FF}=\nu_{23f}^{IN}\\ G_{12f}^{FF}=D_{f}G_{12f}^{IN},\;G_{13f}^{FF}=D_{f}G_{13f}^{IN},\;G_{23f}^{FF}=G_{23f}^{IN} \end{array}$$

Matrix failure:

(10) $$E_{m}^{MF}=D_{m}E_{m}^{IN},\;\nu_{m}^{MF}=\nu_{m}^{IN}$$

where the superscript *IN*, *FF,* and *MF* represent the intact constituents, fiber failure, and matrix failure.

After the damage initiation, the macro properties and stress amplification factors need to be recalculated using RVE models with the degraded constituent properties. When calculating the degraded macro mechanical properties, only the square RVE model is adopted, rather than hexagon RVE, because of its asymmetry in the 2 and 3 directions.

Therefore, the entire multiscale analysis process can be summarized. Once the macro stresses at each integration point of the laminates are obtained by finite element models (FEMs), the micro stresses can be calculated based on Equation (1) via RVEs. The failure of the fiber and matrix should then be examined with Equations (3) and (8) at the reference points in the RVEs separately. If damage does not appear, the calculation will go to the next iteration directly. Otherwise, if any sort of damage occurs, constituent properties should be degraded based on the category of damage, and a new iteration will be executed. The FEM analysis will be finished once a sharp load decrease occurs, which indicates that the whole specimen can no longer sustain the load.

### 2.4. Micro Strength Determination

To get the micro strength of the fiber and matrix in the MMF3 criterion from the macro strength, a back-calculation method with iteration algorithm was implemented. In detail, $T_{f}$ and $C_{f}$ can be calculated with the longitudinal tensile strength $X_{T}$ and compressive strength $X_{C}$ respectively according to Equation (1):

(11) $$\begin{array}{l} \{\begin{array}{c} T_{f}=\max\left(M_{\sigma 11,f}^{\left(i\right)}X_{T}+A_{\sigma 1,f}^{\left(i\right)}\Delta T\right)\\ C_{f}=\max\left(\left|-M_{\sigma 11,f}^{\left(i\right)}X_{C}+A_{\sigma 1,f}^{\left(i\right)}\Delta T\right|\right) \end{array}\\ i=1,2,3\ldots \end{array}$$

where $M_{\sigma 11,f}$ and $A_{\sigma 1,f}$ are the mechanical and thermal stress amplification factors of fiber, as derived in Equation (2), i=1,2,3… denotes the reference points in the fiber.

The micro shear strength $S_{m}$ can be calculated with the macro in-plane shear strength S of the laminates. If only the macro in-plane shear stress ${\bar{\sigma }}_{12}$ exists, which results in the micro shear stress $\sigma_{12}$ and $\sigma_{13}$ according to Equation (2), the matrix failure criterion in Equation (8) can be simplified as:

(12) $$\frac{1}{S_{m}^{2}}\left(\sigma_{12}^{2}+\sigma_{13}^{2}\right)=1$$

So, the back-calculation method of $S_{m}$ is:

(13) $$\begin{array}{l} S_{m}=\sqrt{\max\left[{\left(M_{\sigma 44,m}^{\left(i\right)}S\right)}^{2}+{\left(M_{\sigma 54,m}^{\left(i\right)}S\right)}^{2}\right]}\\ i=1,2,3\ldots \end{array}$$

where $M_{\sigma 44,m}$ and $M_{\sigma 54,m}$ are the mechanical stress amplification factors of matrix, and i=1,2,3… represents the reference points in the matrix.

The micro tensile strength $T_{m}$ and compressive strength $C_{m}$ can be back-calculated from the transverse tensile strength $Y_{T}$ and compressive strength $Y_{C}$ of the matrix. If only exists ${\bar{\sigma }}_{22}$, resulting in the micro stresses $\sigma_{11}$, $\sigma_{22}$, $\sigma_{33}$ and $\sigma_{23}$, the matrix failure criterion in Equation (8) can be simplified as:

(14) $$\left(\frac{1}{T_{m}}-\frac{1}{C_{m}}\right)\left(\sigma_{22}+\sigma_{33}\right)+\frac{1}{T_{m}C_{m}}{\left(\sigma_{22}+\sigma_{33}\right)}^{2}-\frac{1}{S_{m}^{2}}\left[\sigma_{22}\sigma_{33}-\tau_{23}^{2}\right]=1$$

For simplification, 3 parameters are defined as follows:

(15) $$\left\{ \begin{array}{l} \sigma_{I}=\sigma_{22}+\sigma_{33}\\ \sigma_{II}={\left(\sigma_{22}+\sigma_{33}\right)}^{2}\\ \sigma_{III}=\frac{1}{S_{m}^{2}}\left[\sigma_{22}\sigma_{33}-\tau_{23}^{2}\right] \end{array}\right.$$

Then, the Equation (12) can be expressed as:

(16) $$\left(\frac{1}{T_{m}}-\frac{1}{C_{m}}\right)\sigma_{I}+\frac{1}{T_{m}C_{m}}\sigma_{II}-\sigma_{III}=1$$

The solution of $T_{m}$ and $C_{m}$ needs iterative computation. Firstly, the specific value between $C_{m}$ and $T_{m}$ is defined as:

(17) βm=CmTm

When the micro stresses satisfy the failure criterion, Equation (14) can be expressed by $T_{m}$ and $\beta_{m}$:

(18) $$(\sigma_{III}+1)\beta_{m}T_{m}^{2}-(\beta_{m}-1)\sigma_{I}T_{m}-\sigma_{II}=0$$

The positive root of Equation (16) is defined as:

(19) $$\sigma_{eq}=\frac{\left(\beta_{m}-1\right)\sigma_{I}+\sqrt{{\left(\beta_{m}-1\right)}^{2}\sigma_{I}^{2}+4\left(\sigma_{III}+1\right)\beta_{m}\sigma_{II}}}{2\left(\sigma_{III}+1\right)\beta_{m}}$$

When ${\bar{\sigma }}_{22}$ equals the macro transverse tensile strength $Y_{T}$, $\sigma_{eq-T}^{\left(i\right)}$ at each reference point in the matrix can be calculated, and the maximum value is defined as $T_{m,T}$. When ${\bar{\sigma }}_{22}$ equals the macro transverse compression strength $Y_{C}$, $T_{m,C}$ can be defined in a similar way. Since the matrix failure criterion in Equation (8) is applicable under both tension and compression conditions, the micro tensile strength of the matrix that is back-calculated in different cases ($T_{m,T}$ and $T_{m,C}$) should be equal. So, the value of $\beta_{m}$ is iterated in a reasonable range until the difference between $T_{m,T}$ and $T_{m,C}$ is in the tolerance. Then, the correct $T_{m}$ and $C_{m}$ values are obtained.

## 3. Materials and Experiments

In order to evaluate the effectiveness of the proposed multiscale strategy in matrix-dominant failure behaviors, a series of tensile and compressive tests of the unidirectional laminates were conducted at the off-axis angles 10°, 30°, 45°, and 67°. The dimensions of off-axis tensile and compressive specimens are 250 mm × 25 mm × 2 mm and 140 mm × 12 mm × 2 mm respectively. Tabs are applied at the ends to prevent the early failure of the tension specimens due to the clamping pressure and the buckling of the compression specimens. The off-axis tests were carried out at a loading rate 1 mm/min. The composite system T300/5228A is used in the present study. T300 carbon fiber is a reinforcement made by the Toray Company (Tokyo, Japan), and 5228A is a toughened epoxy resin manufactured by the Beijing Aeronautic Material Academe of AVIC (Aviation Industry Corporation of China, Beijing, China). All test specimens were cured in autoclaves at a temperature of 180 °C.

**Figure 2:**
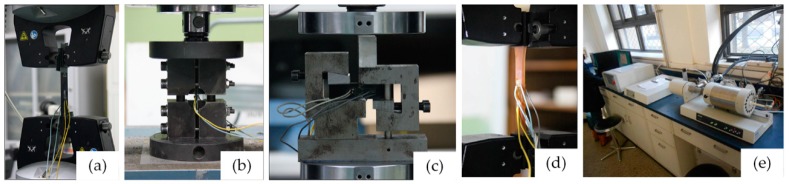
Experiments for basic mechanical properties of the T300/5228A laminates: (**a**) Tension; (**b**) Compression; (**c**) Shear; (**d**) Tension of matrix; (**e**) Thermomechanical analysis of matrix.

**Figure A1:**
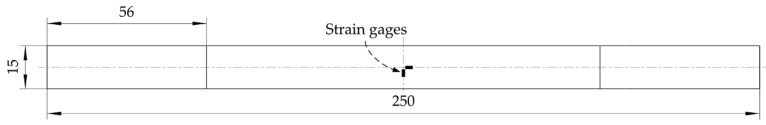
Dimensions of the longitudinal tensile specimens (mm).

**Figure A2:**
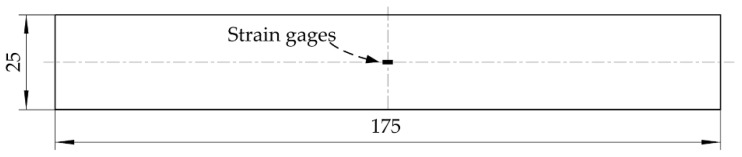
Dimensions of the transverse tensile specimens (mm).

**Figure A3:**
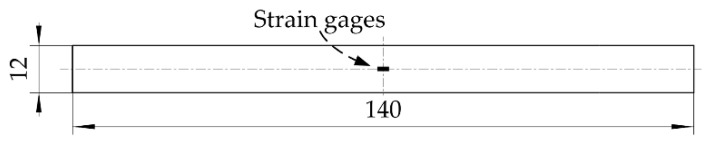
Dimensions of the unidirectional compressive specimens (mm).

**Figure A4:**
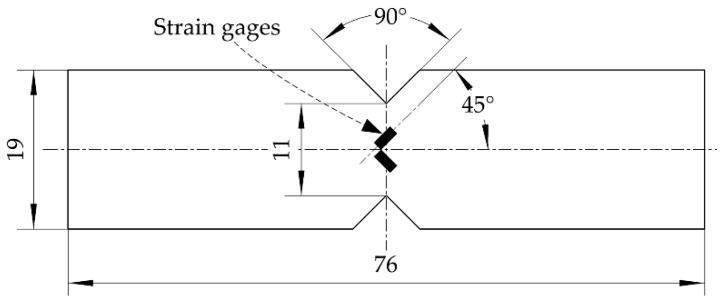
Dimensions of the V-notched shear specimens (mm).

**Figure A5:**
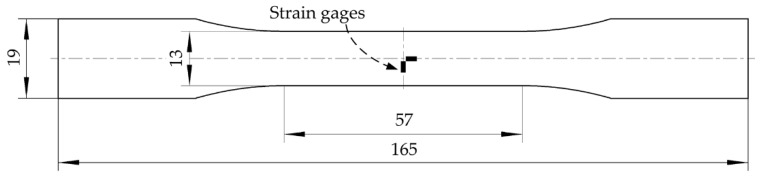
Dimensions of the tensile specimens of the matrix (mm).

The basic mechanical properties of T300/5228A were tested based on the standards of American Society for Testing Materials (ASTM), as shown in Figure 2. Tensile modulus and strength of the unidirectional laminates were tested according to ASTM D3039 [32], and compressive modulus and strength of the unidirectional laminates were tested according to ASTM D6641 [33]. In-plane shear properties of were tested on the laminates with stacking sequence [0/90]_8s_ using the V-notched beam method in ASTM D5379 [34], which can produce a pure shear condition. The tensile modulus of the matrix 5228A was tested according to ASTM D638 [35]. The primary dimensions of the specimens and the stain gages within above basic tests were shown as Figure A1, Figure A2, Figure A3, Figure A4 and Figure A5 in Appendix A.

The longitudinal tensile and compressive tests of the unidirectional laminates were conducted using Instron 8802 (Illinois Tool Works Inc., Norwood, MA, USA), servohydraulic testing system with ±250 kN loading capacity. The transverse tensile and compressive tests of the unidirectional laminates, as well as the tensile test of the matrix, were conducted using Instron 5966 (Illinois Tool Works Inc., Norwood, MA, USA) dual column tabletop frame with ±10 kN loading capacity. As recommended, the loading rate for the above tests was 1 mm/min to provide quasi-static loading conditions. The coefficient of thermal expansion of the matrix was tested using the thermo-mechanical analysis method with the NETZSCH DIL 402C manufactured by NETZSCH Group (Bavaria, Germany) according to ASTM E831 [36].

## 4. Finite Element Models

**Figure 3:**
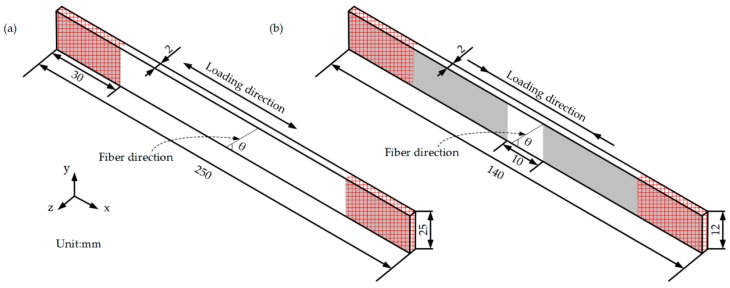
Finite element models: (**a**) Off-axis tension; (**b**) Off-axis compression.

The finite element models (FEMs) of the off-axis tensile and compressive specimens were built with reduced integration continuum solid elements (designated as C3D8R) in ABAQUS 6.13-1. The dimensions of the models are the same as test specimens and the off-axis angle θ are 10°, 30°, 45° and 67°, as shown in Figure 3. The translation freedoms in the y-, z-direction of the nodes on the surfaces with grey shadows were constrained to simulate the clamping effect of the fixtures. Two reference points were coupled with the both end faces of the models respectively in the x-direction. The translation freedom in the x-direction was constrained at one of the reference points. The displacement loading in the x-direction was applied at the other reference point to simulate tension and compression. In order to eliminate the influence of stress concentration caused by loading boundary, the damage is only defined at the region of interest, except at the red highlighted areas near the ends of the models, using subroutine USDFLD of ABAQUS.

## 5. Results and Discussion

The failure modes in the basic tests were acceptable according to the ASTM standards [25,26,27,28,29,30], which ensured the validity of the obtained parameters. In the longitudinal tensile test of the unidirectional laminate, the failure was explosive in the middle gage section. The longitudinal tensile modulus and strength $X_{T}$ were determined as 125.49 GPa and 1762.3 MPa, and the in-plane Poisson’s ratio $\nu_{12}$ was determined as 0.288. As for transverse tension, the failure was lateral in the middle gage section. The transverse modulus and strength $Y_{T}$ were determined to be 8.56 GPa and 71.1 MPa respectively.

In the longitudinal compressive test of the unidirectional laminate, failure occurred in the middle gage section [37]. The longitudinal compressive modulus and strength $X_{C}$ were determined to be 122.32 GPa and 1362.2 MPa. As for transverse compression, the failure was through-thickness at the grip top area. The transverse tensile modulus and strength $Y_{C}$ were determined to be 10.88 GPa and 218.3 MPa. In the V-notched shear tests of the laminate with stacking sequence [0/90]_8s_, the failure happened in the V-notched section. The in-plane shear modulus and strength S were determined to be 4.53 GPa and 83.5 MPa.

The properties of fiber T300 are presented in Table 1. The Young’s modulus $E_{m}$ and Poisson’s ratio $\nu_{m}$ of the matrix 5228A were determined to be 3.22 GPa and 0.346, based on the tensile test of the matrix. The properties of fiber and matrix were used as micro mechanical properties in the RVE models.

Based on the macro strength of laminates obtained from the basic standard tests, the micro strength of the fiber and matrix can be determined using the back-calculation method proposed in the previous Section 2.4. The obtained micro strength, listed in Table 2, has been used to determine the damage initiation in the multiscale analysis of laminates under different off-axis loading conditions.

### 5.1. Failure Analysis of Laminates under Off-Axis Loading

**Figure 4:**
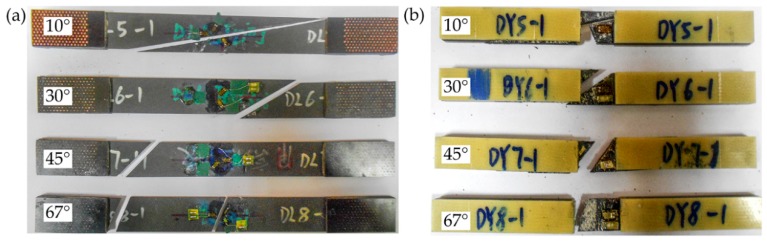
Failure modes in the experiments: (**a**) Off-axis tension; (**b**) Off-axis compression.

**Figure 5:**
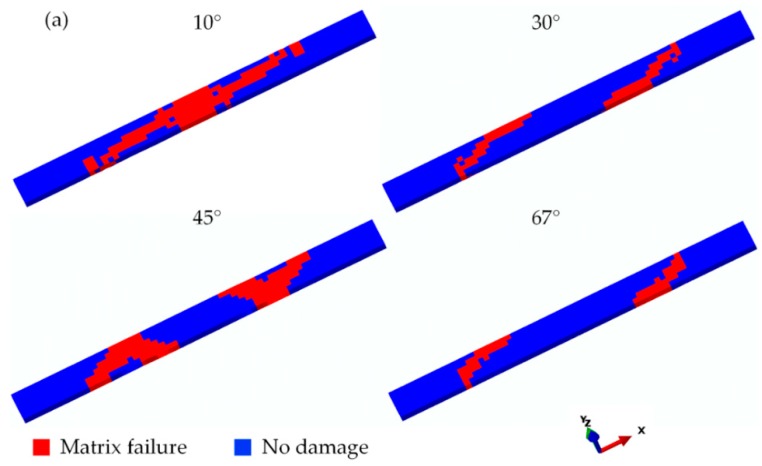

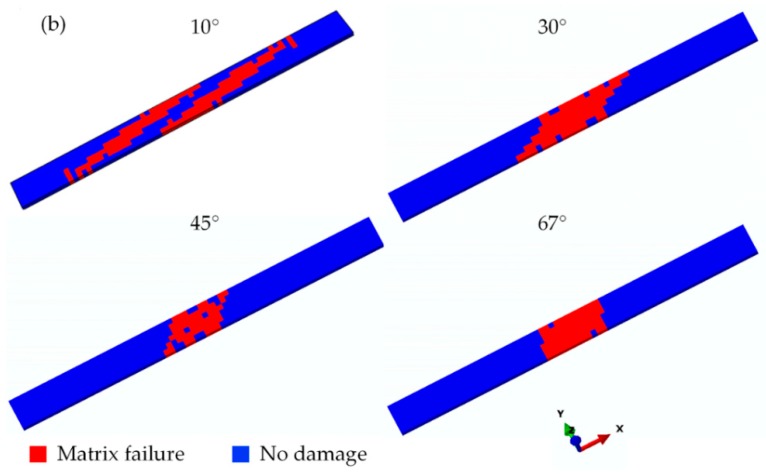
Failure modes in the simulations: (**a**) Off-axis tension; (**b**) Off-axis compression.

With the established multiscale models and back-calculated micro strength, the failure behaviors of unidirectional laminates under different off-axis loading conditions have been simulated. The failure modes in the experiments and simulations of off-axis tension and compression are presented in Figure 4 and Figure 5 respectively. It can be seen that the fracture angles are the same as off-axis angels. As the materials and loading conditions are idealized in the FE models, two fracture zones can be seen in the off-axis tension and compression with small off-axis angle. But only one of these fracture zones appears in reality. Good agreement has been achieved between the experimental and numerical results. Due to the fairly clean fracture edges, the matrix failure can be determined as the dominant mode of the laminates under off-axis loading. Therefore, it is reasonable to judge the accuracy of the proposed multiscale strategy in the matrix-dominant failure definition by the off-axis strength prediction of laminates.

Table 3 presents the off-axis strength of laminates from the established multiscale models and experiments. Based on the MMF3 failure criterion, the predicted results are larger than experimental results for off-axis tension, while they are smaller for compression. Predictions are more accurate for tension tests: the maximum error is 8.52% at off-axis angle 67°. As for off-axis compression, the maximum error is −9.27% at off-axis angle 45°.

**Figure 6:**
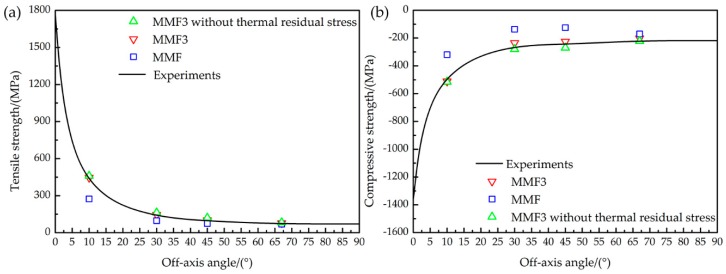
Strength comparison between experimental and numerical results: (**a**) Off-axis tension; (**b**) Off-axis compression.

To quantify the accuracy improvements of MMF3 criterion, the predictions of off-axis strength with MMF criterion are also presented as a benchmark. According to Equations (3) and (4), and reference [28], the calculated micro strength values of the matrix in the MMF criterion are $T_{m}=123.9$ MPa and $C_{m}=237.7$ MPa, while the $T_{f}$ and $C_{f}$ are the same as those of the MMF3 criterion. As the results in Figure 6 show, the curves were obtained by fitting the experimental results. The MMF failure criterion obviously underestimated the off-axis strength, especially at off-axis angles 10°, 30°, and 45°. This indicates that the MMF criterion is not accurate enough in the matrix failure definition of the laminates. In comparison, the revised MMF3 failure criterion shows varying degrees of improvements at different off-axis angles. Hence, the proposed multiscale strategy with the MMF3 criterion can predict the off-axis strength and failure modes of the laminates more effectively.

### 5.2. Effect of Thermal Residual Stress

The influence of thermal effect in the multiscale strategy was explained with RVE analysis. When the effect of thermal residual stress is ignored in the multiscale models, the predicted strength is obviously overestimated according to the results in Figure 6. Especially at the off-axis angle 45°, the off-axis strength without the thermal residual stress is about 10% greater than those that consider the thermal effect. In order to further assess the thermal effect on the analysis of failure behaviors, the left side of Equation (8) can be defined as damage index $K_{M}$ to represent the damage status of matrix. Damage occurs in the matrix when $K_{M}$ equals to 1. Consequently, the difference of damage status with and without the thermal residual stress can be evaluated using the relative deviation as:

(20) $$RD=\frac{K_{{}_{M}}^{0}-K_{{}_{M}}^{\prime }}{K_{{}_{M}}^{0}}\times 100\%$$

where $K_{{}_{M}}^{0}$ is the maximum damage index of matrix considering thermal residual stress, while $K_{{}_{M}}^{\prime }$ is not.

**Figure 7:**
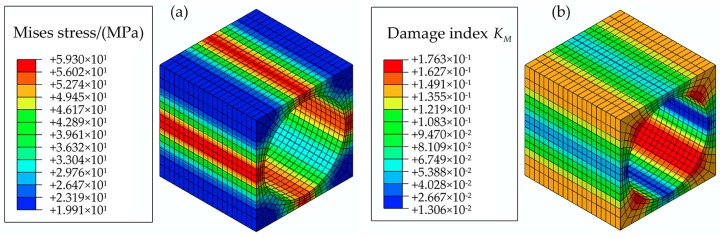
Distributions of Mises stress and damage index in the matrix caused by thermal residual stress: (**a**) Mises stress; (**b**) Damage index.

The square RVE is adopted to obtain the damage status at the constituent level. The distributions of the Mises stress and damage index $K_{M}$ of the matrix without mechanical macro stresses, but only thermal residual stress are presented in Figure 7. The maximum Mises stress and damage index $K_{M}$ caused by the thermal residual stress are about 59 MPa and 0.176. Thus, the effect of thermal residual stress in the multiscale strategy cannot be neglected.

**Figure 8:**
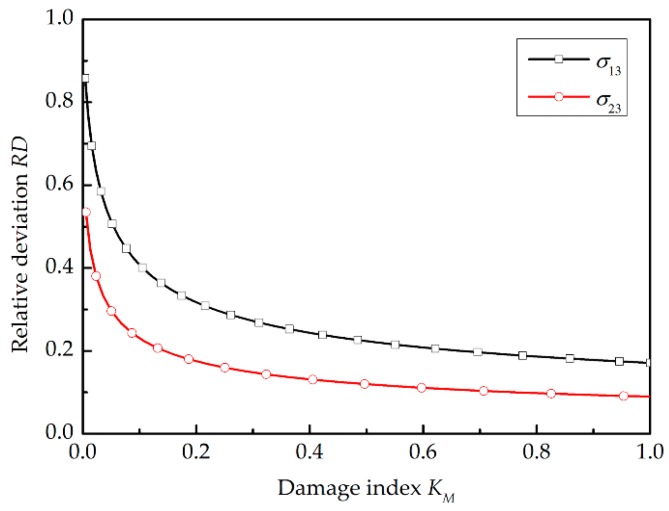
The relative deviation-damage index curve under macro stress $\sigma_{13}$ and $\sigma_{23}$.

The damage status under the macro stresses $\sigma_{13}$, $\sigma_{23}$, and $\sigma_{22}$ has been studied. The curves of relative deviation RD considering thermal residual stress or not are shown with damage index $K_{M}$ in Figure 8. As the damage index $K_{M}$ increases with the load, the RD tends to decrease monotonically until matrix damage occurs. However, until the last moment ($K_{M}=1$), the influence of thermal residual stress did not decrease to a negligible level. At the moment of damage initiation under the macro stresses $\sigma_{13}$ and $\sigma_{23}$, there are still relative deviations of about 10% and 20% respectively. The positive RD means that the matrix failure will lag if the thermal residual stress is neglected. The RD caused by $\sigma_{22}$ is not presented, as it is much smaller than the ones under shear loading. From this perspective, the overestimated off-axis strength of laminates due to the neglected thermal effect can be explained. The thermal residual stress will contribute to the final failure by affecting the matrix damage status.

### 5.3. Sensitivity Analysis of Matrix-Dominant Micro Strength

In order to study the physical meanings of the micro strength in MMF3 criterion and their influence rules on the macro strength, a sensitivity analysis was performed. As the off-axis failure of T300/5228A in the present study are matrix-dominant, the sensitivity analysis focuses on the matrix-dominant strength $T_{m}$, $C_{m}$ and $S_{m}$. The variation ranges are listed in Table 4.

**Figure 9:**
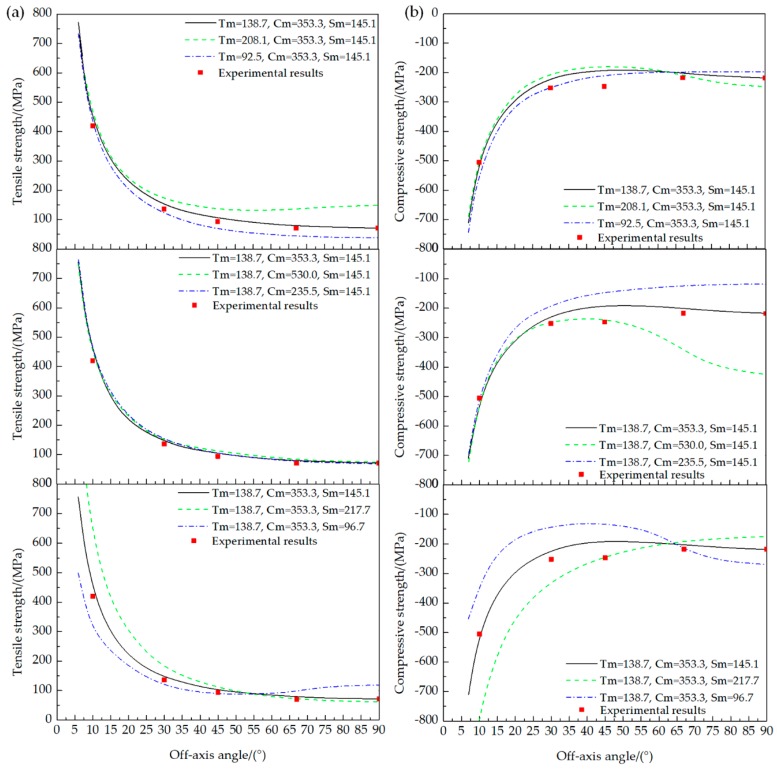
Sensitivity analysis of matrix-dominant micro strength: (**a**) Off-axis tension; (**b**) Off-axis compression.

With different micro strengths of the matrix, the corresponding off-axis strength was obtained using the established multiscale models. Figure 9 shows the relationship between predicted macro off-axis strength and matrix-dominant micro strength.

When there is a 50% increase or decrease of $T_{m}$, the predicted off-axis tensile strength exhibits a significant positive correlation, especially at lager off-axis angles (30°–90°). For the predicted off-axis compressive strength, it is worth noting that three data curves intersect at the off-axis angle 65°. When the off-axis angle is smaller than 65°, the predicted compressive strength (absolute value) gets smaller with an increase of $T_{m}$, while the trend reverses when the off-axis angle is larger than 65°. In this regard, the change of $T_{m}$ will not obviously affect the compressive strength at off-axis angle 65°.

The change of $C_{m}$ has little influence on the off-axis tensile strength, but a great positive influence on the off-axis compressive strength. The positive influence of increasing $C_{m}$ on off-axis compressive strength remarkably increases with the off-axis angle.

The change of $S_{m}$ significantly affects both the off-axis tensile and compressive strength, especially when the off-axis angle is relatively small. For the off-axis tension, the strength increases with $S_{m}$ when the off-axis angle is smaller than 55°, but decreases when the off-axis angle is larger than 55°. For the off-axis compression, a similar trend can be observed, but the intersection point of three data curves was determined to be at the off-axis angle 65°.

The influence rule of the matrix-dominant micro strength in the MMF3 criterion on the macro off-axis strength accords with their physical meanings. $T_{m}$ represents the micro tensile strength of matrix, and it mainly influences the off-axis tensile strength with a relatively large off-axis angle. $C_{m}$, the micro compressive strength of matrix, is closely related to the off-axis compressive strength, with a relatively large off-axis angle. $S_{m}$ represents the micro shear strength of matrix, and dominates when the off-axis angle is relatively small.

## 6. Conclusions

A multiscale strategy has been developed based on the MMF3 criterion. Two kinds of RVEs, square and hexagon, taken as being representative of different fiber distributions were used to determine the quantitative relationships between macro- and the micro- stresses. The micro strength in the MMF3 criterion was back-calculated from the macro strength of laminates. The properties of damaged elements were degraded at the constituent level. Then, the established multiscale models were applied to the failure analysis of composite laminates under different off-axis loading conditions. The predicted results were compared with a series of tests to further assess the effectiveness of the proposed strategy. Based on the present study, the following conclusions can be made:

- Both the test and prediction results suggest that the off-axis loading failure are matrix-dominant. On the one hand, the revised MMF3 failure criterion shows a considerable improvement in the failure definition of the matrix compared with the MMF criterion. On the other hand, the agreements between the test and prediction results demonstrate that the multiscale strategy is effective in matrix-dominant failure analysis of composite laminates.
- If thermal residual stress is neglected in the multiscale analysis, the matrix failure will lag under shear loading, leading to overestimated strength. As the load goes up, the influence of thermal residual stress decreases monotonically, but cannot reach a negligible level before damage initiation.
- The influence rules of matrix-dominant micro strength on the macro off-axis strength coincide with their physical meanings. The micro tensile and compressive strength of the matrix mainly affects the off-axis strength at a relative large off-axis angle, while the micro shear strength of the matrix dominates the off-axis strength at small off-axis angles. The intersection points of the data curves indicate that the micro compressive and shear strength of the matrix have little influence on the macro compressive strength at an off-axis angle 65°, and the macro tensile strength at an off-axis angle 55° is rather insensitive to the micro shear strength of the matrix.

## Figures and Tables

**Table 1:** Properties of fiber T300 [18].

Properties	Values
Longitudinal modulus $E_{1f}$/(GPa)	227.0
Transverse modulus $E_{2f}=E_{3f}$/(GPa)	25.0
In-plane shear modulus $G_{12f}=G_{13f}$/(GPa)	28.0
Out-of-plane shear modulus $G_{23f}$/(GPa)	9.50
In-plane Poisson’s ratio $\nu_{12f}=\nu_{13f}$	0.245
Out-of-plane Poisson’s ratio $\nu_{23f}$	0.316
Fiber volume fraction $V_{f}$	56%

**Table 2:** Micro strength of T300/5228A.

Constituent	Micro strength	Values
Fiber/T300	Tensile strength $T_{f}$/(MPa)	3091.8
	Compressive strength $C_{f}$/(MPa)	2440.6
Matrix/5228A	Tensile strength $T_{m}$/(MPa)	138.7
	Compressive strength $C_{m}$/(MPa)	353.3
	Shear strength $S_{m}$/(MPa)	145.1

**Table 3:** Strength of unidirectional laminates T300/5228A under off-axis loading.

Condition	Off-Axis Tension Strength/(MPa)			Off-Axis Compression Strength/(MPa)		
Angle	Experiment	Prediction	Error	Experiment	Prediction	Error
10°	420.0	446.5	6.32%	505.4	511.2	1.15%
30°	136.0	145.5	6.99%	252.0	231.2	−8.25%
45°	93.7	101.6	8.42%	247.0	224.1	−9.27%
67°	71.9	78.6	8.52%	217.4	201.6	−7.26%

**Table 4:** Variation ranges of the matrix-dominant micro strength for the sensitivity analysis.

Number	$T_{m}/(\mathrm{MPa})$	$C_{m}/(\mathrm{MPa})$	$S_{m}/(\mathrm{MPa})$	Description
1	138.7	353.3	145.1	Reference value
2	208.1	353.3	145.1	$T_{m}$ is increased by 50%
3	92.5	353.3	145.1	$T_{m}$ is decreased by 50%
4	138.7	530.0	145.1	$C_{m}$ is increased by 50%
5	138.7	235.5	145.1	$C_{m}$ is decreased by 50%
6	138.7	353.3	217.7	$S_{m}$ is increased by 50%
7	138.7	353.3	96.7	$S_{m}$ is decreased by 50%

## Appendix A

The following figures show the detailed dimensions of specimens and the strain gages within the tensile, compressive, and shear tests of unidirectional laminates and the tensile tests of the matrix.
